# Validity of the test for attentional performance in neurological post-COVID condition

**DOI:** 10.1038/s41598-025-09128-2

**Published:** 2025-07-07

**Authors:** Susan Seibert, Irina Eckert, Catherine N. Widmann, Taraneh Ebrahimi, Fabian Bösl, Christiana Franke, Harald Prüss, Joachim L. Schultze, Gabor C. Petzold, Omid Shirvani

**Affiliations:** 1 Center for Neurology, University of Bonn Medical Center, Bonn, Germany; 2 German Center for Neurodegenerative Diseases (DZNE), Venusberg-Campus 1, C99, 53127 Bonn, Germany; 3 Department of Neurology and Experimental Neurology, Charité- Universitätsmedizin Berlin, Berlin, Germany; 4 German Center for Neurodegenerative Diseases (DZNE), Berlin, Germany; 5 Genomics and Immunoregulation, Life & Medical Sciences (LIMES) Institute, University of Bonn, Bonn, Germany

**Keywords:** COVID-19, Post-/Long-COVID, Cognitive impairment, Neuropsychological assessment, Test battery, Complex attention, Psychology, Fatigue, Neurology

## Abstract

Neurological post-COVID condition (PCC) often involves attentional deficits that impact daily functioning. Traditional paper-based tests, like the Trail-Making Test (TMT), may inadequately capture these impairments due to their short duration and dependence on numerical and alphabetic sequencing. This study evaluates the validity of three subtests of the computerized Test for Attentional Performance (TAP) as alternatives for detecting attentional impairments in PCC. In the ongoing NEURO LC-19 DE study, 108 subjects aged 18 to 79 years, with PCC-related cognitive complaints (*n* = 67, 73% f) and healthy controls (*n* = 41, 56% f) underwent neuropsychological testing. The prevalence of impairment and classification ability of the TAP subtests were evaluated alongside standard paper-based tests, including the TMT and Montreal Cognitive Assessment, using receiver operating characteristic (ROC) analysis and regression. The TAP subtests identified significant impairments in sustained attention and processing speed in one-third of PCC patients, surpassing traditional tests in sensitivity, and classifying PCC with an AUC of 78%. Omissions in sustained attention significantly differentiated groups (OR = 1.14, *p* = 0.016, 95% CI [1.02–1.26]). Fatigue correlated with poorer performance on speed and accuracy (*r* > 0.30, *p* < 0.05). Cognitive slowing is prevalent in neurological PCC but is scarcely captured by conventional assessments. The TAP’s computerized format with automated norming and independence from alphanumeric stimuli shows promise in improving the discriminatory ability for identifying attentional deficits in PCC patients.

**Supplementary Information:**

The online version contains supplementary material available at 10.1038/s41598-025-09128-2.

## Introduction

Post-COVID Condition (PCC) is a widely known sequelae of SARS-CoV-2 infection, affecting different body systems. It refers to a condition characterized by symptoms that persist for at least three months following infection and cannot be ascribed to other diagnoses^1^. Among the most common symptoms are fatigue, attention and memory difficulties as well as a blurry cognitive state referred to as “brain fog”^2^. The prevalence of cognitive impairment varies widely across studies, with rates ranging from 5 to 25%^3–5^. This discrepancy is often due to methodological heterogeneity, as demonstrated by a recent scoping review^6^.

The exact mechanisms by which the virus induces persistent impairment of cognitive function remain unclear. Recent research suggests various potential explanations, such as vascular involvement, blood-brain barrier disruption, systemic inflammation, and metabolic disturbances^7,8^. Nonetheless, the underlying pathomechanisms are still under scientific investigation and remain ambiguous^9–11^. Due to cognitive impairment, many patients with neurological PCC are often referred to neurological outpatient clinics, where they frequently undergo neuropsychological evaluations to uncover their subjective cognitive complaints. Neuropsychology is vital in understanding brain fog in PCC, revealing cognitive and emotional challenges that guide effective assessment and rehabilitation strategies^12^.

Results from several meta-analyses indicate that attention and executive functions (EF) are among the cognitive domains most often impacted in this population, manifesting as the so-called dysexecutive syndrome^2,13–15^. Specific attentional impairments following COVID-19 were evident as early as the day of hospital discharge, as demonstrated in a previous study utilizing computerized measures of reaction time (RT) and sustained attention^16^. Even six to nine months after infection, individuals report impaired alertness and exhibit a reduced ability to sustain attention for even brief periods of three minutes compared to healthy controls^17,18^. Attention serves as a foundational cognitive process upon which all other cognitive tasks depend, making it indispensable for effective functioning in daily life. Impairments in attention can, therefore, disrupt the integration and execution of complex cognitive functions, leading to difficulties in personal, occupational, and social activities. Consequently, the assessment of attention in individuals with PCC is crucial, as disruptions in this core function may underpin broader neuropsychological deficits. Prior studies described a specific processing speed impairment in PCC patients, often manifested as general cognitive slowing that significantly affects tasks such as driving or working^19–21^. One explanation for this core feature is the “hypoarousal model” of the brain, proposing a link between fatigue and cognitive deficits due to reduced activity of the networks responsible for maintaining alertness^22^. It has been shown that subjective cognitive symptoms (e.g., memory or concentration difficulty) are associated with a reduction of RT, making RTs a valid indicator to assess cognitive impairment in both patients and healthy individuals^23,24^.

Within the commonly performed neuropsychological assessments, a variety of cognitive tests can be administered to detect such deficits in attention and alertness. Widely used are screening instruments like Montreal Cognitive Assessment (MoCA) and standard paper-pencil tests, such as the Trail Making Test (TMT) or the D2 Test of Attention. These assessments evaluate sub-functions of attention but may not always detect subtle impairment^16,25^. Recent advancements in computerized and remote assessments, prompted in part by the COVID-19 pandemic, have led clinicians to adopt newer cognitive tests for several study populations^26–28^. Digital cognitive assessments offer automated scoring and normative data, which in turn reduces rater errors and inter-rater variability^29^. Moreover, sub-domains such as sustained and divided attention, which are challenging to evaluate with traditional paper-based tests, can be more accurately measured with digital tools^16^. Additionally, assessments of sustained attention are recognized for their high ecological validity^30,31^.

One alternative to paper-pencil assessment is the Test for Attentional Performance (TAP), which evaluates specific attention functions^31^. It is available in 18 languages and does not necessitate fine motor skills or numerical comprehension. However, despite their increasing use, computerized test batteries do not consistently demonstrate an advantage. Arbula et al.^25^ compared three measures from the web-based Toolbox of Attention Control with standard neuropsychological assessments. Their findings revealed that the variables Flanker accuracy and Visual Arrays performance from the computerized tasks significantly contributed to differentiating between PCC and the control group. However, the experimental tasks did not surpass the standard tests in sensitivity, possibly due to limitations in the data analysis method or the variability of cognitive symptoms in the PCC group. Consistently, a recent meta-analysis by Velichkovsky et al.^32^ emphasized the need for more rigorously validated computerized tools.

In light of these findings, this study seeks to address this gap by evaluating the validity of the TAP in detecting specific attentional-executive impairments in PCC patients, and comparing its effectiveness to traditional paper-based assessments. In doing so, we aim to contribute to the development of reliable digital methods for assessing cognitive function in PCC patients.

## Methods

The data analysed in this clinimetric study were collected as part of the ongoing observational longitudinal study “Neurological manifestations of Long COVID-19 in Germany” (NEURO LC-19 DE), led by the German Center for Neurodegenerative Diseases (DZNE) (https://neurocov.eu). Subjects included in this study visited the outpatient clinics for Post-COVID at the University Hospital of Bonn or the Charité Universitätsmedizin Berlin for study participation from October 2023 to July 2024. They had to present with subjective cognitive complaints that had persisted for at least three months post-infection. All patients underwent a comprehensive physical consultation, including a detailed anamnesis (e.g., symptoms, medical history), a neurological examination (e.g., assessment of motor and sensory function, reflexes, coordination), and a blood draw (e.g., inflammatory markers, blood count) to confirm neurological PCC according to criteria from the National Institute for Health and Care Excellence (NICE)^1^. A spinal tap (cerebrospinal fluid analysis) was performed optionally. The healthy control group (CTL) consisted of individuals who had a previous confirmed SARS-CoV-2 infection but no ongoing symptoms including cognitive complaints; hence, not meeting NICE criteria for PCC. Exclusion criteria were pregnancy/breastfeeding, participation in a drug trial, absence of confirmed SARS-CoV-2 infection by polymerase chain reaction test or SARS-CoV-2 antibodies, and an acute infection within the preceding four weeks. Only adult (≥ 18 years) subjects fluent in German were included. Subjects were recruited via media (e.g., local newspaper) announcements. All sociodemographic data and medical history were recorded in the DZNE study database during the initial anamnesis.

All data was stored in pseudonymized form. Participants provided written informed consent before being enrolled in the study. The research was conducted in accordance with the Helsinki Declaration. The study was approved by the Ethics Committee of the University of Bonn (081/23-EP) and registered at the German Clinical Trials Register (DRKS00032475).

### Baseline Measures

All participants underwent a comprehensive neuropsychological assessment covering the major cognitive domains memory, attention, language, visuospatial, and executive functions. In addition, self-report questionnaires were used to measure levels of fatigue and depression, subjective memory perception and general health. A full list of tests and questionnaires administered is available in Supplementary Table S1. All participants first completed the cognitive screening, followed by the paper-pencil and computerized cognitive assessments and lastly completed the self-report questionnaires. The Reliable Digit Span, based on the Wechsler Adult Intelligence Scale-IV (WAIS-IV) subtest digit span, was applied as an embedded performance validity measure (PVT)^33^.

### Montreal cognitive assessment

The MoCA is a brief global cognitive screening tool developed for the detection of mild cognitive impairment and dementia^34^. It consists of seven subdomains that are commonly impaired in the target population, including visuospatial/executive abilities, naming, attention, language, abstraction, memory, and orientation. The total score ranges from 0 to 30, with the traditional cut-off for impairment being set at < 26^34^. Recent literature has highlighted that this original cut-off may lead to a heightened rate of false positives^35^. Newer studies suggest that a more accurate cut-off for detecting cognitive impairment in specific populations, including those with (Post-)COVID-19-related cognitive deficits, may lie between < 23 and < 24^36,37^. Our primary analyses use the traditional cut-off; in addition to that, we have provided analyses with the adjusted cut-off (< 24) in the supplements (Supplementary Table S2). The MoCA takes approximately ten minutes to administer. At baseline, the German Version C was administered.

### Trail making test

The TMT contains two subparts, both administered on paper^38^. The TMT-A requires individuals to connect circled numbers from 1 to 25 in ascending order. In the TMT-B, the task is to switch between digits in ascending order and letters in sequence of the alphabet when drawing the lines (e.g., 1-A-2-B-3-C etc.). Both parts should be completed as fast as possible, as the time is being tracked. There is a time limit of three and five minutes to complete, respectively. While TMT-A primarily assesses information processing speed, TMT-B requires mental flexibility and is the most commonly used test for divided attention and EF^5^. Normative data by Tombaugh (2004) were applied to convert raw TMT scores into age- and education-adjusted z-scores^39^.

### Test of attentional performance

The TAP is a computerized test battery that comprises a collection of tests covering different domains of attention^31^. In this study, three subtests of the TAP (version 2.3.1) were administered: alertness, sustained attention and divided attention. In the *alertness* subtest, participants are required to press a button as quickly as possible in response to a cross displayed on the screen. The RTs (median), representing *intrinsic alertness*, are recorded by the system. In a second condition, the cross is preceded by a tone. Subjects are asked to ignore the cue and respond to the cross, indicating *phasic alertness*. The total duration of this subtest is five minutes.

The *sustained attention* subtest comprises a 15-minute stream of 450 individually presented geometric figures that differ in their features (shape, color, filling). The test subjects have to press the reaction key when they recognize two figures with the same shape in immediate succession. The primary outcome variable is the number of omission errors.

The *divided attention* subtest integrates attention via the visual and auditory modalities, which are presented in parallel for three minutes. The visual task consists of recognizing when four small crosses are arranged next to each other on the display (4 × 4) so that they form a square. Simultaneously, the participants must identify when the high and low tones played are repeated (two high or two low). When they detect one of the 33 critical stimuli, they have to press the key. Again, the number of omission errors is the main variable of interest.

All performance measures, including RTs and errors, are reported by the system in raw scores and age-normed T-scores (M = 50, SD = 10).

### Self-report questionnaires

#### Fatigue severity scale (FSS)

The FSS is a self-rating questionnaire for assessing the severity of fatigue symptoms^40^. It contains nine items about the fatigue level in the past week. Responses are rated on a 7-point Likert scale ranging from 1 (“strongly disagree”) to 7 (“strongly agree”). The total score can fall between 9 and 63. An average score is derived from the total score with values ranging from 1 to 7, whereby a score of four or higher is considered indicative of problematic fatigue. The German version of the scale was adapted by Valko et al.^41^ and shows high internal consistency and reliability with Cronbach’s α = 0.93.

#### Becks depression inventory-II (BDI-II)

The BDI-II is one of the most widely used questionnaires to screen for depressive symptoms (Beck, Steer, and Brown 1996; German version: Hautzinger, Keller, and Kühner 2006). It comprises 21 item groups, each item is rated on a 4-point scale ranging from 0 to 3. The items cover domains such as sadness, crying, and sleep disturbance. The total score of the BDI-II can range from 0 to 63 points and can be classified into four categories, although it is not intended for clinical diagnosis. A score of 13 or less is not indicative of depression, scores between 14 and 19 suggest mild depression, scores between 20 and 28 indicate moderate symptomatology, and scores greater than 29 reflect severe symptoms. The Cronbach’s alpha for internal consistency ranges from α = 0.74 to 0.94.

### Statistical analysis

A power analysis was conducted in advance using G*Power 3.1.9.7^44^ to determine the adequacy of the sample size. The results indicated that a sample of *n* = 102 would be required to achieve 80% power for detecting a medium effect (*d* = 0.05) with a significance criterion of α = 0.05 group comparisons.

For descriptive statistics, we compared patients with PCC and controls using Student`s t-test for continuous variables and chi-square tests (χ^2^) for categorical variables. All assumptions were checked before conducting analyses. Outliers in cognitive measures (± 3 SD from the mean) were winsorized^45^. For baseline cognitive performance comparisons, raw scores adjusted for age and days since infection, were analyzed using one-way analysis of covariance (ANCOVA). Where applicable, z or T norm scores were reported, with cognitive impairment defined as scores below − 1.5 SD. For comparison purposes, the impairment rate was also calculated using a cut-off of -1 SD. Effect sizes were reported as Cohen’s *d*, phi-coefficient (*φ*), or partial eta squared (*η*_*p*_^2^*)*. A binomial logistic regression analysis was conducted to evaluate the ability of neuropsychological tests measuring attention, corrected for sex and years of education, to classify individuals with PCC and controls. Linearity was assessed using the Box-Tidwell procedure^46^. To avoid multicollinearity, the predictor phasic alertness was excluded from further analyses due to its high correlation with intrinsic alertness (*r* = – 0.917), as both variables measure RT. Unless otherwise specified, all predictors were included in the model as continuous variables to preserve the full range of measurement. Results were reported in a forest plot as odds ratios (OR) with 95% confidence intervals (CIs). To address the age difference between PCC participants and controls, we conducted a matched subsample analysis using a ± 5-year tolerance for both the cognitive test comparison and the logistic regression model. The results are presented in Supplementary Tables S3 and S4. Receiver operating characteristic (ROC) analyses, including the area under the curve (AUC) and the Youden Index (*J*), were performed to determine sensitivity and specificity. AUCs of TAP and TMT were compared using DeLongs test. Pearson correlations for the total sample were computed between subjective fatigue, depression ratings, and cognitive performance. An α-level of *p* < 0.05 was set for statistical significance and adjusted accordingly for Bonferroni correction. The statistical analyses were conducted using IBM SPSS Statistics (version 27.0) and R Statistical Software (version 4.2.1).

## Results

### Baseline characteristics

Of the 110 subjects recruited between October 2023 and July 2024, two (1.8%) withdrew from the study. The remaining 108 participants, aged 18 to 79 years, including 72 (66.7%) females, were included into our analysis. All participants passed the PVT. Basic demographic and clinical characteristics of the participants are detailed in Table 1.

The CTL group was younger and had a lower prevalence of past psychiatric disorders compared to the PCC group. Most participants had a university degree (52.7%) and were in a partnership (57.3%). The most common pre-existing comorbidity in the total sample was allergies (51.9%). In the PCC group, chronic pulmonary disorders, such as asthma (22.4%) were predominant, whereas arterial hypertension was more common in the CTL group (12.2%).

Only PCC participants (*n* = 7) were hospitalized during infection, most of them on a monitoring ward or intensive care unit (57.14%). The most frequent symptom during acute COVID-19 in PCC participants was cough (70.1%), while in healthy controls it was sore throat (80.5%). The most common self-reported symptoms currently experienced by individuals with PCC are fatigue (97.0%), memory and concentration difficulties (94.0%), and word-finding deficits (74.6%). Due to the persistence of symptoms, 22.4% of patients were temporarily unable to work at the time of the baseline visit.

**Table 1:** Key demographic and clinical characteristics.

Baseline characteristics	PCC	CTL	Total			
	*n* = 67	*n* = 41	*n* = 108	***t***	***p***	***d***
	***M*** **(SD)**					
Age (years)	47.16 (13.00)	35.90 (15.27)	42.89 (14.89)	− 4.09	**< 0.001**	− 0.81
Education (years)	16.31 (2.52)	16.78 (2.55)	16.49 (2.53)	0.93	0.354	0.18
Body Mass Index (kg/m)^2^	25.93 (5.29)	25.37 (4.54)	25.82 (5.35)	− 0.56	0.575	− 0.11
Time since infection (days)	294.74 (36.28)	619.02 (284.41)	781.77 (300.05)	− 2.79	**0.006**	− 0.56
	***n (%)***			***χ2***	***p***	***φ***
Sex (% female)	49 (73.10)	23 (56.10)	72 (66.70)	3.32	0.068	0.18
Premorbid psychiatric condition	23 (34.30)	4 (9.80)	27 (25.00)	8.19	**0.004**	0.28

CTL = Healthy controls; PCC = Post-COVID Condition. Cohens *d* and phi-coefficient indicate effect sizes: small (*d* ≥ 0.2; *φ* ≥ 0.1), moderate (*d* ≥ 0.5; *φ* ≥ 0.3), or large (*d* ≥ 0.8; *φ* ≥ 0.5) effects. Significant results after Bonferroni correction (*p* < 0.008) are indicated in bold.

### Neuropsychological profile

**Fig. 1:**
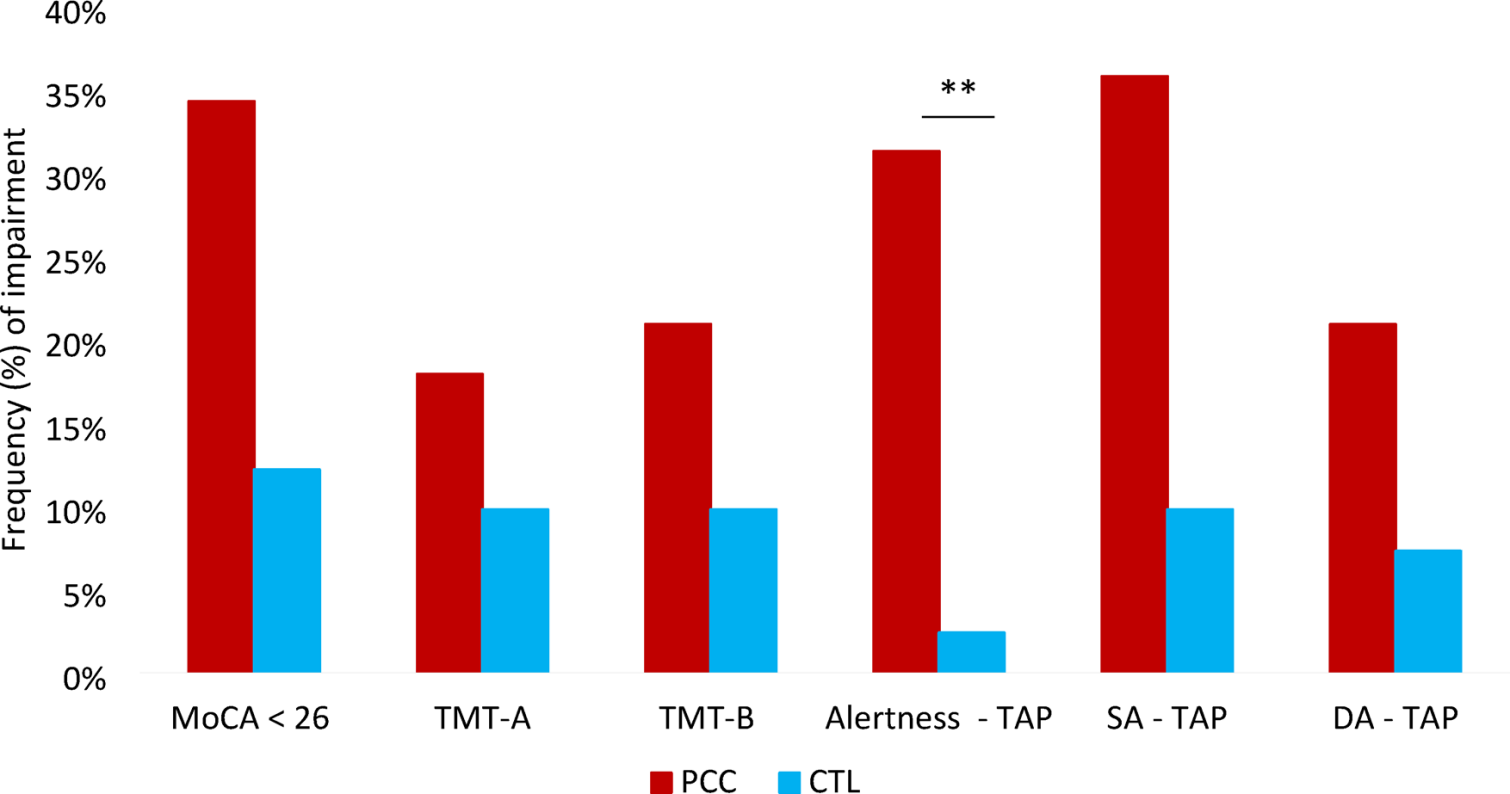
Frequencies of cognitive impairment (− 1.5 SD). CTL = Healthy controls; DA = divided attention; MoCA = Montreal Cognitive Assessment; PCC = Post-COVID Condition; SA = sustained attention; TAP = Test for Attentional Performance; TMT = Trail-Making-Test. Results of chi-square tests with Bonferroni adjustment (*p* < 0.008); ** *p* < 0.01.

**Fig. 2:**
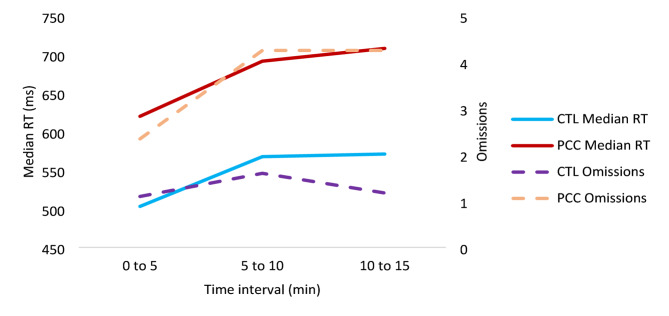
Sustained attention subtest of the TAP. CTL = Healthy controls; PCC = Post-COVID Condition; RT = reaction time; TAP = Test for Attentional Performance. Median RTs and interquartile ranges (IQR: Q1-Q3) for PCC and CTL across three 5-minute intervals. PCC: 0–5 min., IQR = (485–665) ms; 5–10 min., IQR = (532–813) ms; 10–15 min., IQR = (529–816) ms; CTL: 0–5 min., IQR = (473–564) ms; 5–10 min., IQR = (486–616) ms; 10–15 min., IQR = (478–600) ms; PCC patients consistently conducted more omissions (all *p* < 0.05) and the CTL group demonstrated a significantly faster median RT throughout the time span (all *p* < 0.01).

The mean neuropsychological test results, adjusted for age and time since infection, reveal that the CTL group consistently exhibited lower rates of cognitive impairment (defined as scores below the cut-off) and demonstrated better overall performance on neuropsychological tests compared to PCC patients (Table 2; Fig. 1). At the total sample level, the MoCA’s mean score was 26.78 (2.15), indicating no cognitive impairment. Approximately one-third of patients exhibited substantial cognitive slowing and a decline in concentration over time, illustrated by the increase in reaction time *F*(1,105) = 16.37, *p* < 0.001, *η*_*p*_^*2*^ = 0.137, and a higher number of omission errors (i.e., greater decline in accuracy) in sustained attention *F*(1,105) = 13.47, *p* < 0.001, *η*_*p*_^*2*^ = 0.116, compared to control subjects (see Fig. 2). While the number of omission errors in the divided attention task showed only a small difference between groups, with PCC participants making, on average, 1.52 more errors, this difference was statistically significant, *F*(1,105) = 8.21, *p* = 0.005, *η*_*p*_^*2*^ = 0. 074. However, the two groups did not differ in TMT-A, *F*(1,105) = 7.20, *p* = 0.009, *η*_*p*_^*2*^ = 0.065 or TMT-B, *F*(1,105) = 4.11, *p* = 0.045, *η*_*p*_^*2*^ = 0.038, after applying Bonferroni correction. The frequency of cognitive impairment using a more liberal cut-off of -1 SD is presented in Supplementary Figure S1. Using this cut-off, approximately 50% of patients and 12 to 20% of controls show cognitive impairment in the alertness subtest, which assesses RT.

According to the established criterion that cognitive domain impairment requires below-cutoff performance on two tests^47^16.4% (*n* = 11) of patients and 7.3% (*n* = 3) of controls exhibited impairments in attention, as measured by both TMT-A and B. Considering only computerized attention tests, 34.4% (*n* = 23) of PCC patients and one healthy control (2.4%) displayed cognitive impairment. An interesting pattern emerged when both paper-and-pencil and computerized measures were analyzed together: 23.9% (*n* = 16) of patients demonstrated impaired performance on the computer-based measures despite normal TMT scores, whereas only 10.4% (*n* = 7) showed impairments on both test types. Furthermore, impairments were noted in TMT-A and TMT-B alone, with no corresponding deficits in computerized attention tests, only in 6% (*n* = 4) of patients and 7.3% (*n* = 3) of controls. The demographic characteristics of these subgroups are demonstrated in the supplementary description.

Comparing domain-specific performance with the results of the MoCA reveals that 22.4% (*n* = 15) of patients scored above 26 on the screening yet demonstrated impairment in either paper-based tests, computerized tests, or both. This was also true for 7.3% (*n* = 3) of healthy controls.

**Table 2:** Baseline cognition and questionnaire results.

		PCC (*n* = 67)	CTL (*n* = 41)			
		M ^a^ (SE)	M ^a^ (SE)	F	*p*	η_p_^2^
Neuropsychological test						
MoCA		26.28 (0.25)	27.55 (0.33)	8.23	**0.005**	0.074
TMT-A, sec		33.21 (1.42)	26.61 (1.85)	7.20	0.009	0.065
TMT-B, sec		69.07 (2.91)	58.83 (3.80)	4.11	0.045	0.038
TAP						
Intrinsic alertness, ms		340.98 (14.73)	237.54 (19.23)	16.37	**< 0.001**	0.137
Phasic alertness, ms		325.51 (12.88)	235.20 (16.82)	16.31	**< 0.001**	0.137
Sustained attention, omissions		10.75 (1.02)	4.21 (1.34)	13.47	**< 0.001**	0.116
Divided attention, omissions		2.79 (0.30)	1.27 (0.40)	8.21	**0.005**	0.074
Self-report questionnaires						
FSS ^b^		5.79 (0.14)	2.23 (0.18)	216.83	**< 0.001**	0.678
BDI-II ^c^		15.13 (0.80)	4.10 (1.04)	63.20	**< 0.001**	0.380

CTL = Healthy controls; MoCA = Montreal Cognitive Assessment (cut-off < 26); PCC = Post-COVID Condition; TAP = Test for Attentional Performance; TMT = Trail-Making-Test.

Partial eta squared indicates effect sizes: small *η*_*p*_^*2*^ ≥ 0.01; medium *η*_*p*_^*2*^ ≥ 0.06; large *η*_*p*_^*2*^ ≥ 0.14. Significant results after Bonferroni correction (*p* < 0.006) are indicated in bold.

^a^ means adjusted for age and time since infection *(p* - values are based on group comparisons of adjusted means).

^b^ FSS = Fatigue Severity Scale (cut-off ≥ 4).

^c^ BDI-II = Becks Depression Inventory-II ( *n* = 106, cut-off > 13).

### Classification ability of neuropsychological tests

**Fig. 3:**
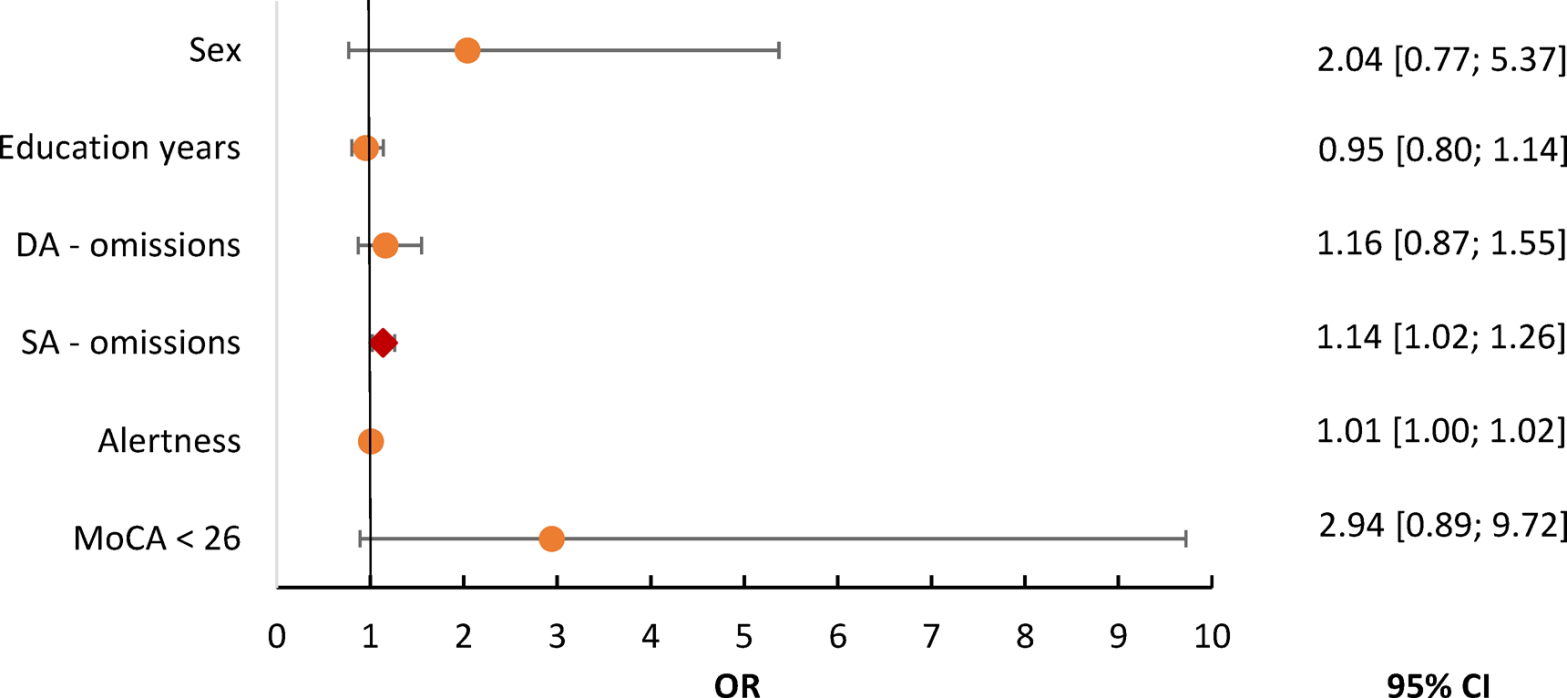
Odds Ratios from logistic regression for PCC classification. Alertness = RT (TAP); CI = Confidence Interval; DA = divided attention (TAP); MoCA = Montreal Cognitive Assessment; OR = Odds Ratio; PCC = Post-COVID Condition; SA = sustained attention (TAP). The variable sex was coded binary (0 = male, 1 = female). Rhombus (red) indicates significant result (*p* < 0.05), while circles (orange) represent non-significant results.

For the binomial logistic regression, we used group as the dependent variable and demographic characteristics (sex, education years) as covariables. The four cognitive test variables that were significantly different between groups were included as input variables. This resulted in a statistically significant regression model *χ²*(6) = 37.00, *p* < 0.001, with a moderate amount of explained variance, as indicated by Nagelkerke`s R^2^ = 39.5% ^48^. In the logistic regression analysis predicting group membership, the sustained attention (omissions) score was the only significant variable, with an OR of 1.14, *p* = 0.016, 95% CI [1.02–1.26], indicating that more omissions on this task were associated with increased odds of being classified in the PCC group. The MoCA cut-score, alertness, divided attention, sex, and education years were not independently associated with group classification (results in Fig. 3 and Supplementary Table S5). The model’s overall classification accuracy was 69.4%, with a sensitivity of 74.6% and a specificity of 61.0%. This resulted in a positive predictive value of 75.7% and a negative predictive value of 59.5%.

**Fig. 4:**
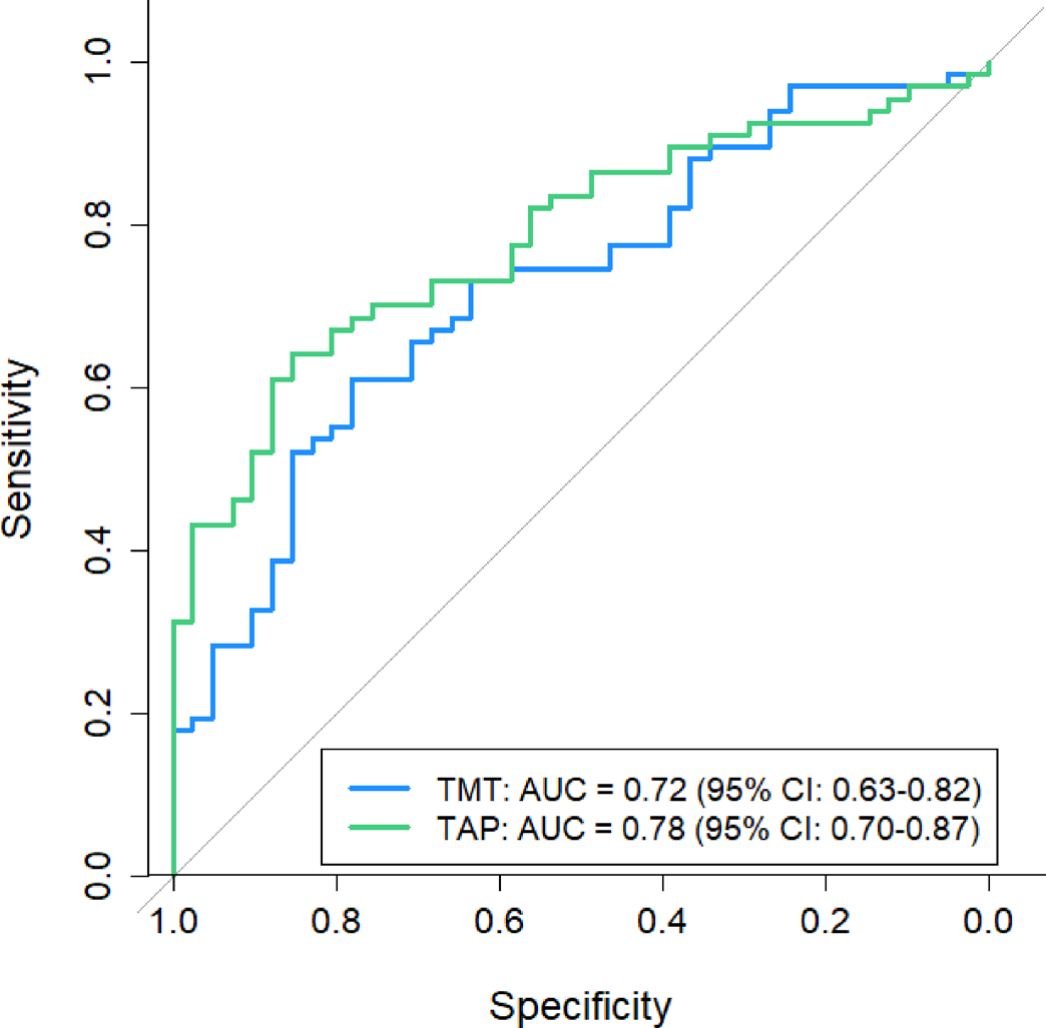
ROC Curves of TMT and TAP. AUC = Area under the Curve; CI = Confidence Interval; TAP = Test for Attentional Performance; TMT = Trail-Making-Test.

To evaluate the discriminative ability of the neuropsychological tests, ROC curves were generated using the predicted probabilities from each test. The AUC values for each predictor are shown in Table 3. The lowest sensitivity was observed with the MoCA, using the cut-off of < 26 to indicate impairment (34.3%), while the highest was noted for omissions in the sustained attention task (77.6%). The RT measure of alertness resulted in the highest specificity of 92.7%. The paper-based attention tests yielded an AUC of 0.72, whereas the computerized tests achieved a higher AUC of 0.78 (see Fig. 4). A one-sided DeLong test comparing both AUCs (H_1_: TAP > TMT) revealed no difference (*p* = 0.122) given the current cohort size. However, it is noteworthy that the computerized tests approached strong discriminative ability, while the AUC of the paper-based tests remained in the moderate range. For a more detailed visualization, the separate ROC curves for each subtest are depicted in the Supplementary Figure S2, which allows for a clearer comparison of their individual discriminative performances.

**Table 3:** Results of receiver operating characteristic analyses.

Predictor	AUC	95% CI	Youden Index (J)	Optimal cut-off	Sensitivity	Specificity
MoCA < 26	0.61	[0.53; 0.69]	0.22	N/A	0.34	0.88
TMT-A, sec	0.72	[0.62; 0.82]	0.37	30.5	0.58	0.81
TMT-B, sec	0.70	[0.60; 0.80]	0.35	58.5	0.67	0.68
Alertness, ms	0.71	[0.62; 0.81]	0.43	289	0.51	0.93
SA, omissions	0.76	[0.67; 0.85]	0.41	3.5	0.78	0.63
DA, omissions	0.68	[0.58; 0.78]	0.26	2.5	0.40	0.85

Optimal cutoffs were determined via Youden Index. Youden Index, *J* = Sensitivity + Specificity − 1, good (*J* > 0.5), moderate (0.3 < *J* > 0.5), or poor (*J* < 0.3) discriminative ability. AUC = Area under the Curve; CI = Confidence Interval; DA = divided attention; MoCA = Montreal Cognitive Assessment; SA = sustained attention; TMT = Trail-Making-Test.

### Correlations

**Fig. 5:**
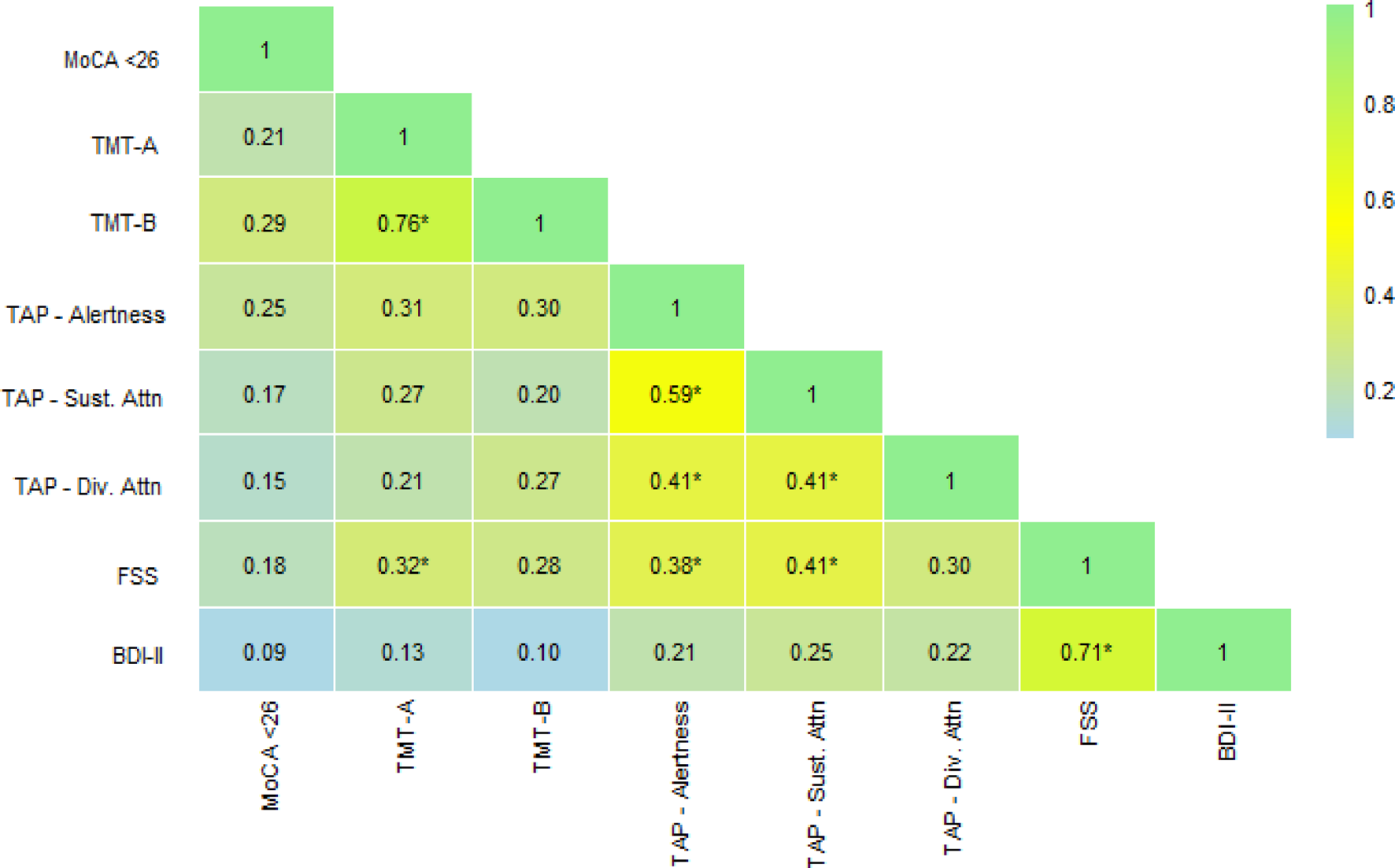
Heatmap of Bonferroni-adjusted Pearson-correlations. BDI-II = Becks Depression Inventory-II; FSS = Fatigue Severity Scale; MoCA = Montreal Cognitive Assessment; TAP = Test for Attentional Performance; TMT = Trail-Making-Test. Asterisks (*) denote significance at *p* < 0.05.

Correlation analyses between self-reported fatigue, mental health, and cognitive outcomes revealed moderate associations between fatigue severity and cognitive test performance, with higher severity linked to slower performance and a higher number of errors (Fig. 5). The strongest correlations with fatigue were observed for the omissions on the sustained attention task (*r* = 0.41, *p* < 0.05), RT (*r* = 0.38, *p* < 0.05), and TMT-A (*r* = 0.32, *p* < 0.05), indicating a time-dependent decline in cognitive function among patients with fatigue. Depressive symptoms, as measured by the BDI-II, were associated with neither the computerized tests (*r* > 0.20, *p* > 0.05) nor the MoCA or paper-pencil tests (*r* < 0.14, *p* > 0.05), yet remained highly associated with fatigue (*r* = 0.71, *p* < 0.05).

## Discussion

The global spread of SARS-CoV-2 has resulted in a significant proportion of patients experiencing prolonged cognitive deficits^49,50^particularly in sustained attention and increased RT^16^. With the emergence of PCC, routine assessment of these specific attentional subdomains has become indispensable^51–53^. Given the high prevalence of fatigue in PCC, frequent in-person neuropsychological assessments are often impractical. Hence, digital assessments or remote tools for tele-assessment could serve as ecologically valid and economically viable instruments for evaluating attentional-executive dysfunctions in PCC^19^.

Our analysis aimed to validate the use of the computerized TAP for digital assessment of attentional functions in this patient population. Based on comprehensive neuropsychological assessments of over 100 participants from our observational study NEURO LC-19 DE, we found significant group differences in cognitive screening (MoCA) and specific attention tests (TAP), though not in TMT-A and TMT-B, which aligns with findings from Ariza and colleagues^54^. Our PCC patients demonstrated a pronounced slowness in RT and increased numbers of omissions in sustained and divided attention tasks. This overall slowness is consistent with previous results by Ortelli et al.^28^ and Becker et al.^55^and supports the hypothesis of brain hypoarousal, particularly within frontoparietal networks^19,56,57^.

Our analysis revealed that only approximately 16% of patients exhibited attentional-executive impairments as assessed by the TMT- A and B, compared to a higher percentage on the TAP, with over one-third of PCC patients performing below 1.5 SD. It is still uncommon for neuropsychological clinicians and researchers to incorporate computerized assessments routinely. For instance, an online survey by Schild et al.^58^ found that in German memory clinics, TMT-A is the sole measure of attention in nearly 40% of institutions, whereas the TAP is employed in only 7%. Our findings highlight the risk of neglecting patients with impairments in sustained attention and processing speed due to the discriminatory differences of both tests.

The optimal neuropsychological assessment methods for patients with PCC have yielded heterogeneous findings across studies. Almeria et al.^59^demonstrated that TMT-A, TMT-B, Stroop test, and Symbol Digit Modalities Test (SDMT) are sensitive in differentiating between healthy controls and PCC patients. Comparably, Ariza et al.^54^ identified the MoCA, SDMT, and phonemic fluency tasks as effective for discrimination. This lack of homogeneous results could be due to different cut-off criteria for impairment and the heterogeneous definitions of PCC and CTL groups, complicating comparisons across studies. Further, a considerable number of our patients (22–30%, depending on the cutoff) scored within the normal range on the MoCA despite exhibiting impairments in attention, echoing concerns raised by Schild et al.^60^ and Lynch et al.^61^ about its validity. Thus, screening tools alone should not be relied upon as the sole measure of impairment in this population^60,62^.

Our regression model identified the number of omissions on the sustained attention task as the single significant predictor for group membership, supporting its clinical utility. The AUCs of the TMT and TAP were comparable. However, our results indicate that the number of omissions in the sustained attention task has the highest sensitivity (78%) for identifying PCC, surpassing other tests. The RT measure (alertness) also yielded the highest specificity (93%). These findings underscore the value of integrating traditional and computerized methods into test batteries for patients affected by PCC, as the latter can reveal nuanced impairments during complex tasks of increased duration^24,63,64^. This integration aligns with the proposed harmonization levels (HL) 2 and HL3, developed by the NeuroCOVID International Neuropsychology Taskforce^65^which aims to standardize post-acute cognitive assessments to enhance test administration and automated scoring. The use of the TAP has also been recommended by the European Network to Improve Diagnosis, Treatment, and Healthcare for Patients with Persistent Somatic Symptoms (EURONET-SOMA) as an appropriate tool for assessing attentional deficits in individuals with PCC^66^.

Several alternatives to the TAP, such as NeuroScreen, NIH Toolbox Cognition, Test My Brain, and the Wiener Test System, are available, providing clinics with additional tablet-based options. Researchers have even developed free online RT measures that patients can complete at home, providing a more accessible assessment option^21^.

Another important finding of our study is the observed correlation between fatigue and cognitive performance in attention, aligning with findings from earlier work^3,56,59^. Fatigue was reported by 91% of our patients, a notably higher prevalence compared to other studies, which have reported rates as low as 30% (for review, see Ceban et al.^3^). This discrepancy may be attributed to our study’s focus on patients with subjective cognitive complaints^67^. Our results confirm the findings of Ortelli et al.^28^who reported a moderate correlation between sustained attention and both the FSS and BDI-II. Similarly, others have found that fatigue is associated with a higher number of errors on attention tasks, although the correlation strength varied^68,69^. These results suggest that the TAP may be useful for quantifying subjective fatigue. However, Zhao et al.^21^ found no relationship between self-reported fatigue, depression, and RT, indicating that these factors alone may not fully explain the variance in performance on attention tasks^70^. Furthermore, we observed a high prevalence of depressive symptomatology among our patients, consistent with literature indicating that psychiatric symptoms may arise or exacerbate after SARS-CoV-2-infection^71^. Depression is often accompanied by cognitive disturbances and fatigue, indicating potential interconnections in both phenotype and pathophysiology between depression and PCC, raising the possibility that depression could, in some cases, be considered a post-viral condition^64,72,73^.

Beyond its diagnostic utility, the TAP framework could inspire the development of similar tasks designed for cognitive training purposes in clinical settings. However, to date, no studies have evaluated its efficacy for patients with PCC. Nevertheless, one study has demonstrated its validity in assessing pre- and post-changes in attention following PCC psychosomatic rehabilitation^64^. Additionally, the TAP holds potential for use in PCC as an embedded performance validity measure, particularly by using RT as an indicator of insufficient effort. However, further research is necessary to validate its applicability to this population^74,75^. Moreover, investigations of increased intra-individual variability (IIV) of RTs in computerized measures will be crucial for the longitudinal assessment of attentional deficits in PCC. In follow-up assessments, IIV could provide valuable insights into the stability of attentional performance, enhancing our understanding of cognitive dynamics in this population, as the progression of attentional deficits is still unclear^54^.

The results of our analysis should be interpreted with several limitations in mind. First, our PCC participants voluntarily presented with subjective cognitive deficits at our clinics. This introduces an ascertainment bias in two ways: severely affected individuals who were bedridden or unable to attend outpatient appointments were excluded; in addition the sample may overrepresent individuals with preserved or increased insight into their cognitive difficulties. Second, the control group differed from patients in certain baseline characteristics, such as age, which may have introduced confounding effects. To address this, we adjusted for key confounders (age and time since infection) and performed a case-control matching analysis using a ± 5-year age tolerance. Of note, the initial absence of matching recruitment reflects the demographic profile of individuals affected by the condition. This is illustrated by the composition of our patient group, which is comprised of roughly two-thirds women, a demographic previously associated with an increased risk of developing PCC^56,76^. Importantly, current literature does not indicate significant sex differences in attentional performance^70^. Therefore, this imbalance is unlikely to bias the results of our validation analysis. Furthermore, the limited sample size may have contributed to the absence of finding a statistically significant difference between the AUCs of the tests. Future validation in a larger cohort could help clarify whether a significant difference exists.

## Conclusion

Cognitive slowing and impairments in sustained attention are frequently observed in neurological PCC. The TAP provides results comparable to paper-pencil tests, showing a tendency for improved discriminatory performance while benefiting from the advantages of computerized cognitive assessment. This enables rapid and reliable identification of deficits in PCC patients. Future research should aim to validate additional computerized tests to identify the most effective selection for cognitive assessment batteries in patients with PCC.

## Electronic supplementary material

Below is the link to the electronic supplementary material.

Supplementary Material 1

## Data Availability

The raw data are subject to the General Data Protection Regulation of the European Union. Requests for data access can be directed to the corresponding author.
