# Correction: Validity of the test for attentional performance in neurological post-COVID condition

**DOI:** 10.1038/s41598-025-18191-8

**Published:** 2025-09-18

**Authors:** Susan Seibert, Irina Eckert, Catherine N. Widmann, Taraneh Ebrahimi, Fabian Bösl, Christiana Franke, Harald Prüss, Joachim L. Schultze, Gabor C. Petzold, Omid Shirvani

**Affiliations:** 1 Center for Neurology, University of Bonn Medical Center, Bonn, Germany; 2 German Center for Neurodegenerative Diseases (DZNE), Venusberg-Campus 1, C99, 53127 Bonn, Germany; 3 Department of Neurology and Experimental Neurology, Charité-Universitätsmedizin Berlin, Berlin, Germany; 4 German Center for Neurodegenerative Diseases (DZNE), Berlin, Germany; 5 Genomics and Immunoregulation, Life & Medical Sciences (LIMES) Institute, University of Bonn, Bonn, Germany

Correction to: *Scientific Reports* 10.1038/s41598-025-09128-2, published online 07 July 2025

In the original version of this Article, Fig. 3 did not display correctly. The original Figure 3 and accompanying legend appear below.

**Fig. 3:**
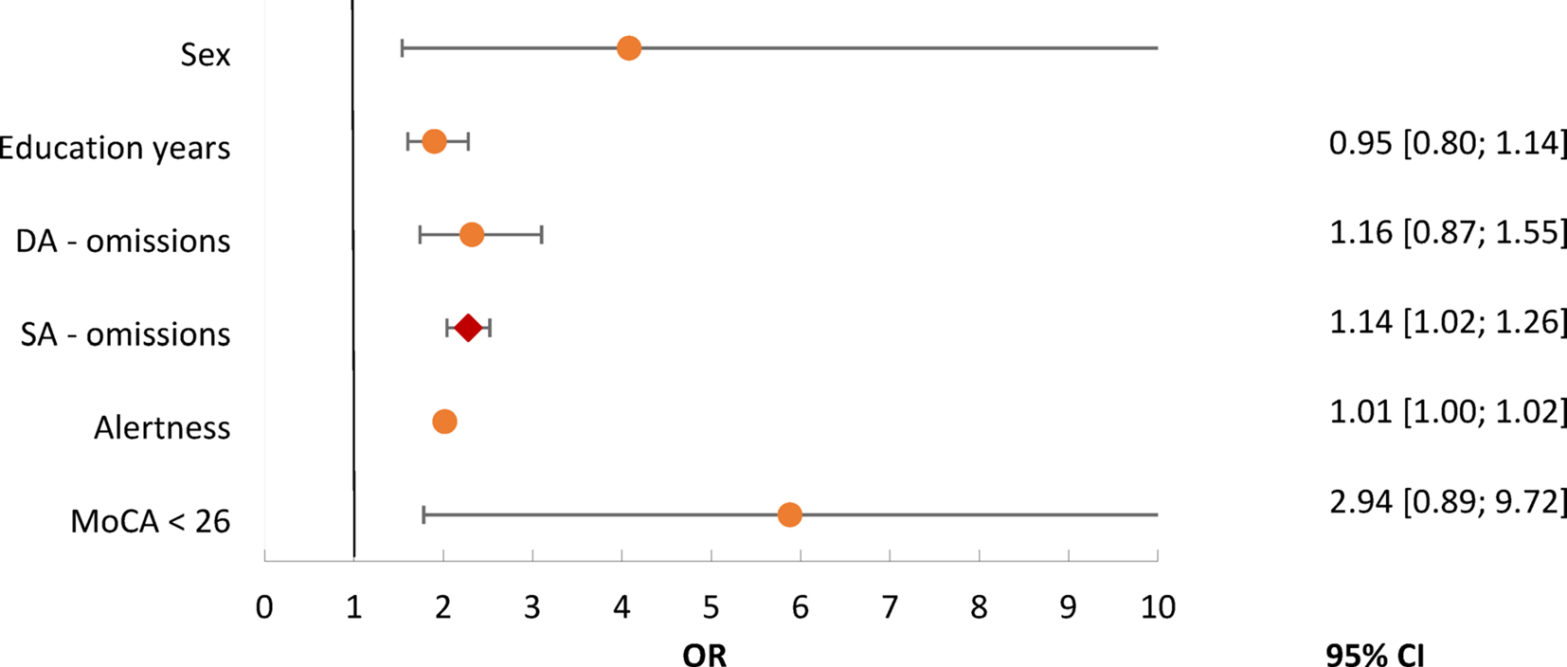
Odds Ratios from logistic regression for PCC classification. Alertness = RT (TAP); CI = Confidence Interval; DA = divided attention (TAP); MoCA = Montreal Cognitive Assessment; OR = Odds Ratio; PCC = Post-COVID Condition; SA = sustained attention (TAP). The variable sex was coded binary (0 = male, 1 = female). Rhombus (red) indicates significant result (*p* < 0.05), while circles (orange) represent nonsignificant results.

The original Article has been corrected.

