# Supplementary material for: Validity of the test for attentional performance in neurological post-COVID condition
Source: Sci Rep. 2025 Jul 7;15:24208. doi: 10.1038/s41598-025-09128-2 (PMC12234741; doi:10.1038/s41598-025-09128-2)
Supplement: Supplementary file 1 — Supplementary Material 1 [file 41598_2025_9128_MOESM1_ESM.pdf]

Supplementary Information: Validity of the Test for Attentional Performance in Neurological Post-COVID Condition

Authors: Susan Seibert, M.Sc.*[1,2], Irina Eckert, M.Sc.[3], Dr. phil. Catherine N. Widmann[1,2], Dr. med. Taraneh Ebrahimi[1,2], Dr. med. Fabian Bösl[3,4], Dr. med. Christiana Franke[3], Prof. Dr. med. Harald Prüß[3,4], Prof. Dr. med. Joachim L. Schultze[2,5], Prof. Dr. med. Gabor C. Petzold[1,2], Dr. med. Omid Shirvani[1,2]

[1]University of Bonn Medical Center, Center for Neurology, Bonn, Germany

[2]German Center for Neurodegenerative Diseases (DZNE), Bonn, Germany

[3]Department of Neurology and Experimental Neurology, Charité-Universitätsmedizin Berlin, Berlin, Germany

[4]Charité Center for Neurology, Neurosurgery and Psychiatry, Berlin, Germany

[5]Genomics and Immunoregulation, Life & Medical Sciences (LIMES) Institute, University of Bonn, Bonn, Germany

***Correspondence**: M.Sc. Susan Seibert

German Center for Neurodegenerative Diseases (DZNE), Bonn, Germany

Venusberg-Campus 1, C99

53127 Bonn, Germany

Susan.Seibert@dzne.de

Phone: (+49) 0228-28757421

Supplementary Table S1 List of complete cognitive assessment and questionnaires at baseline

**Cognitive assessments**
**1.** ***Global cognitive screening*** – MoCA Version C
**2.** ***Verbal learning and memory*** – VLMT Version A
**3.** ***Visual learning and memory*** – BVMT-R Version 1
**4.** ***Working memory*** – WAIS-IV subtest digit span
**5.** ***Attention*** – TAP (Alertness, sustained attention [form], divided attention)
**6.** ***Logical thinking*** – LPS 50+ subtest 3
7. ***Attention/EF –*** TMT A and B
**8.** ***Language –*** RWT semantic and lexical fluency (animals, S-words)
**9.** ***Verbal IQ –*** WST
**Self-report questionnaires**
**10.** SF-36
**11.** CFS
**12.** FSS
**13.** MMQ
**14.** PROMIS 29 Profile v2.1
**15.** PROMIS 4a cognitive function short form
**16.** BDI-II

Note. The full assessment takes approximately 2.5 hours.
BDI-II = Becks Depression Inventory-II; BVMT-R = Brief Visuospatial Memory Test-Revised; CFS = Chalder Fatigue Scale; FSS = Fatigue Severity Scale; LPS = Leistungsprüfsystem; MMQ = Multifactorial Memory Questionnaire; MoCA = Montreal Cognitive Assessment; PROMIS = Patient-Reported Outcomes Measurement Information System; RWT = Regensburger Wortflüssigkeitstest; SF-36 = Short Form-36 Health Survey; TAP = Test for Attentional Performance; TMT = Trail Making Test; VLMT = Verbal Learning and Memory Test; WAIS-IV = Wechsler Adult Intelligence Scale IV; WST = Wortschatztest.

Supplementary Analysis with MoCA cut-off <24

Reconsidering the MoCA cut-off of <24 with improved specificity, as suggested by several authors, we found impairment in n = 9 (13.4%) PCC patients and one (2.4%) CTL subject. This distribution of impaired scores did not differ between the groups, $\chi^2 = 1.92$, $p = 0.166$. Comparing attentional domain-specific performance with the results of the MoCA reveals that 29.8% (n = 20) of patients scored above 24 on the screening yet demonstrated impairment in either paper-based tests (n = 3, 4.4%), computerized tests (n = 12, 17.9%), or both (n = 5, 7.5%). This was also true for 9.7% (n = 4) of healthy controls. A new logistic regression model, including MoCA <24 as a predictor, with group as the dependent variable was significant $\chi^2(6) = 34.31$, $p < 0.001$, explaining a moderate amount of variance (Nagelkerke`s R^2 = 37.0%). The sustained attention variable (omissions) remained the only statistically significant variable (see Table S3, OR = 1.14, $p = 0.017$, 95% CI [1.02 - 1.26]). The model's overall classification accuracy was 71.3%, with a sensitivity of 76.1% and a specificity of 63.4%. For the MoCA <24 cutoff, the ROC curve revealed an AUC of 0.555 [95% CI: 0.445 – 0.664] with a sensitivity of 13.4% and a specificity of 97.6%. The Youden Index for the MoCA <24 was 0.11, which reflects an overall poor discriminative ability of the test at this threshold.

Supplementary Table S2 Results of binomial logistic regression with adjusted MoCA cut-off

	B	SE	*p*	OR	95% Confidence Interval (CI)	
					Lower	Upper
**Sex**	0.677	0.488	0.166	1.98	0.76	5.12
**Education years**	-0.036	0.091	0.692	0.96	0.81	1.15
**MoCA (<24)**	0.946	1.224	0.439	2.58	0.23	28.34
**Alertness**	0.009	0.005	0.093	1.01	1.00	1.02
**SA - omissions**	0.125	0.052	**0.017***	1.13	1.02	1.26
**DA - omissions**	0.161	0.147	0.273	1.17	0.88	1.56

Note. The model includes an intercept term (B = -2.781, SE = 2.011, Wald = 1.91, p = 0.167).
Alertness = Reaction time (TAP); CI = Confidence Interval; DA = divided attention; MoCA = Montreal Cognitive Assessment; OR = Odds Ratio; SA = sustained attention. The variable sex was coded binary (0 = male, 1 = female). * $p < 0.05$ (bold)

Analysis with age-matched CTL sample

To address the age difference between the PCC and CTL groups for the comparison of baseline cognition and questionnaire results (see Table S2) as well as for the logistic regression analysis (Table S3), a matched sample was created using a ±5-year tolerance (CTL n = 27). The distributions between the matched groups did not differ (Kolmogorov-Smirnov, p >0.05). The results of the Mann-Whitney-U test indicated that there was no significant difference in median age between the PCC (*Mdn* = 49, IQR = 17) and CTL (*Mdn* = 38, IQR = 31), U = 715.50, Z = -1.58, p = 0.115.

Supplementary Table S3 Baseline cognition and questionnaire results with age-matched CTL sample.

	**PCC** (*n* = 67)	**CTL** (*n* = 27)			
Neuropsychological test	***Mdn* (IQR)**	***Mdn* (IQR)**	***U***	***p***	***r***
MoCA	26 (3)	28 (3)	514.5	**<0.001**	-0.34
TMT-A, sec	32 (14)	25 (10)	523.0	**<0.001**	-0.33
TMT-B, sec	67 (29)	51 (17)	535.0	**0.002**	-0.32
TAP					
Intrinsic alertness, ms	292 (165)	244 (31)	557.0	**<0.001**	-0.30
Phasic alertness, ms	268 (126)	247 (43)	570.5	**0.005**	-0.29
Sustained attention, omissions	7 (13)	2 (5)	369.0	**<0.001**	-0.44
Divided attention, omissions	2 (3)	1 (2)	594.0	0.008	-0.27
Self-report questionnaires	***Mdn* (IQR)**	***Mdn* (IQR)**	***U***	***p***	***r***
FSS	6 (1.5)	2 (0.9)	41.5	**<0.001**	-0.74
BDI-II	15 (8)	3 (4)	139.5	**<0.001**	-0.66

Note. Becks Depression Inventory-II (cut-off > 13); CTL = Healthy controls; FSS = Fatigue Severity Scale (cut-off ≥ 4); MoCA = Montreal Cognitive Assessment; PCC = Post-COVID Condition; TAP = Test for Attentional Performance; TMT = Trail-Making-Test. Pearson correlation coefficient indicates effect sizes: small $r \geq 0.1$; medium $r \geq 0.3$; large $r \geq 0.5$. Significant results after Bonferroni correction ($p < 0.006$) are indicated in bold.

Supplementary Table S4 Binomial logistic regression (age-matched sample) for classification of PCC.

	B	SE	*p*	OR	95% Confidence Interval (CI)	
					Lower	Upper
**Age**	0.014	0.021	0.499	1.01	0.97	1.06
**Sex**	0.866	0.592	0.143	2.38	0.75	7.58
**Education years**	-0.166	0.111	0.133	0.85	0.68	1.05
**MoCA**	0.613	0.720	0.395	1.85	0.45	7.57
**Alertness**	0.006	0.005	0.260	1.01	1.00	1.02
**SA - omissions**	0.131	0.064	**0.042***	1.14	1.01	1.10
**TMT-A**	0.007	0.046	0.881	1.01	0.92	1.10
**TMT-B**	0.020	0.025	0.428	1.02	0.97	1.07

Note. The model includes an intercept term (B = -1.399, SE = 2.546, Wald = 0.30, p = 0.583).
Alertness = Reaction time (TAP); CI = Confidence Interval; DA = divided attention; MoCA = Montreal Cognitive Assessment; PCC = Post-COVID Condition; OR = Odds Ratio; SA = sustained attention. The variable sex was coded binary (0 = male, 1 = female). * $p < 0.05$ (bold)

The binomial logistic regression model with the age-matched sample was significant, $\chi^2(8) = 31.93$, $p < 0.001$, with a moderate amount of explained variance, as indicated by Nagelkerke`s R^2 = 41.2%. Even after accounting for age, sex, and education and including the five cognitive scores that differed between groups, sustained attention (omissions) remained the only significant predictor of group membership.

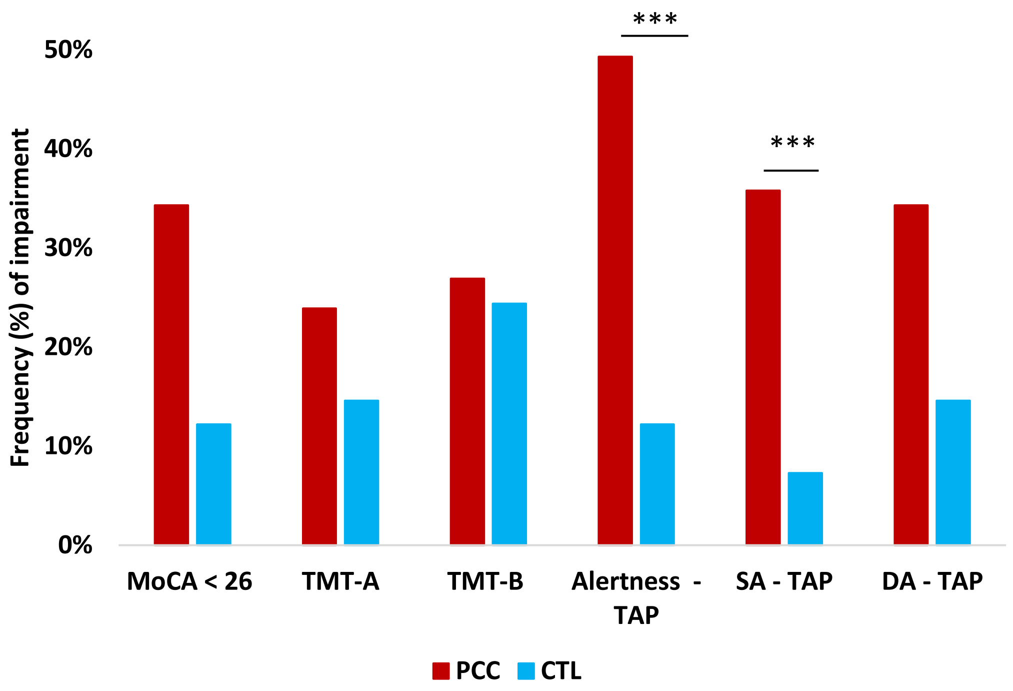

Supplementary Fig. S1 Frequencies of cognitive impairment (-1 SD).
CTL = Healthy controls; DA = divided attention; MoCA = Montreal Cognitive Assessment; PCC = Post-COVID Condition; SA = sustained attention; TAP = Test for Attentional Performance; TMT = Trail-Making-Test.
Results of chi-square tests with Bonferroni adjustment ($p < 0.008$); *** $p < 0.001$

Supplementary description – subgroups of impairment

In the group with impairments on computerized tests and normal performance on paper tests, there were no differences from the rest of the sample in terms of age: $t(106) = -0.62$, $p = 0.538$, $d = -1.63$, sex, $\chi^2(1) = 0.87$, $p = 0.350$, $\varphi = 0.090$, or years of education: $t(106) = -0.80$, $p = 0.426$, $d = -0.211$. However, this group contained a significantly higher proportion of PCC participants compared to control subjects, $\chi^2(1) = 8.82$, $p = 0.003$, $\varphi = 0.286$. Interestingly, in the group with impairments on TMT-A and B, but intact performance on computerized tests, there were no significant differences in any of the characteristics (age, $t(106) = -0.31$, $p = 0.759$, $d = -0.120$, education years, $t(106) = 1.62$, $p = 0.107$, $d = 0.635$, sex, $\chi^2(1) = 0.31$, $p = 0.580$, $\varphi = -0.053$, or group membership, $\chi^2(1) = 0.08$, $p = 0.783$, $\varphi = -0.027$).

Supplementary Table S5 Results of binomial logistic regression

	B	SE	*p*	OR	95% Confidence Interval (CI)	
					Lower	Upper
**Sex**	0.712	0.494	0.150	2.04	0.77	5.37
**Education years**	-0.049	0.091	0.589	0.95	0.80	1.14
**MoCA (<26)**	1.078	0.610	0.077	2.94	0.89	9.72
**Alertness**	0.008	0.005	0.104	1.01	1.00	1.02
**SA - omissions**	0.129	0.054	**0.016***	1.14	1.02	1.26
**DA - omissions**	0.145	0.148	0.327	1.16	0.87	1.55

Note. The model includes an intercept term (B = -2.637, SE = 2.016, Wald = 1.69, p = 0.193).
Alertness = Reaction time (TAP); CI = Confidence Interval; DA = divided attention; MoCA = Montreal Cognitive Assessment; OR = Odds Ratio; SA = sustained attention. The variable sex was coded binary (0 = male, 1 = female). * $p < 0.05$ (bold)

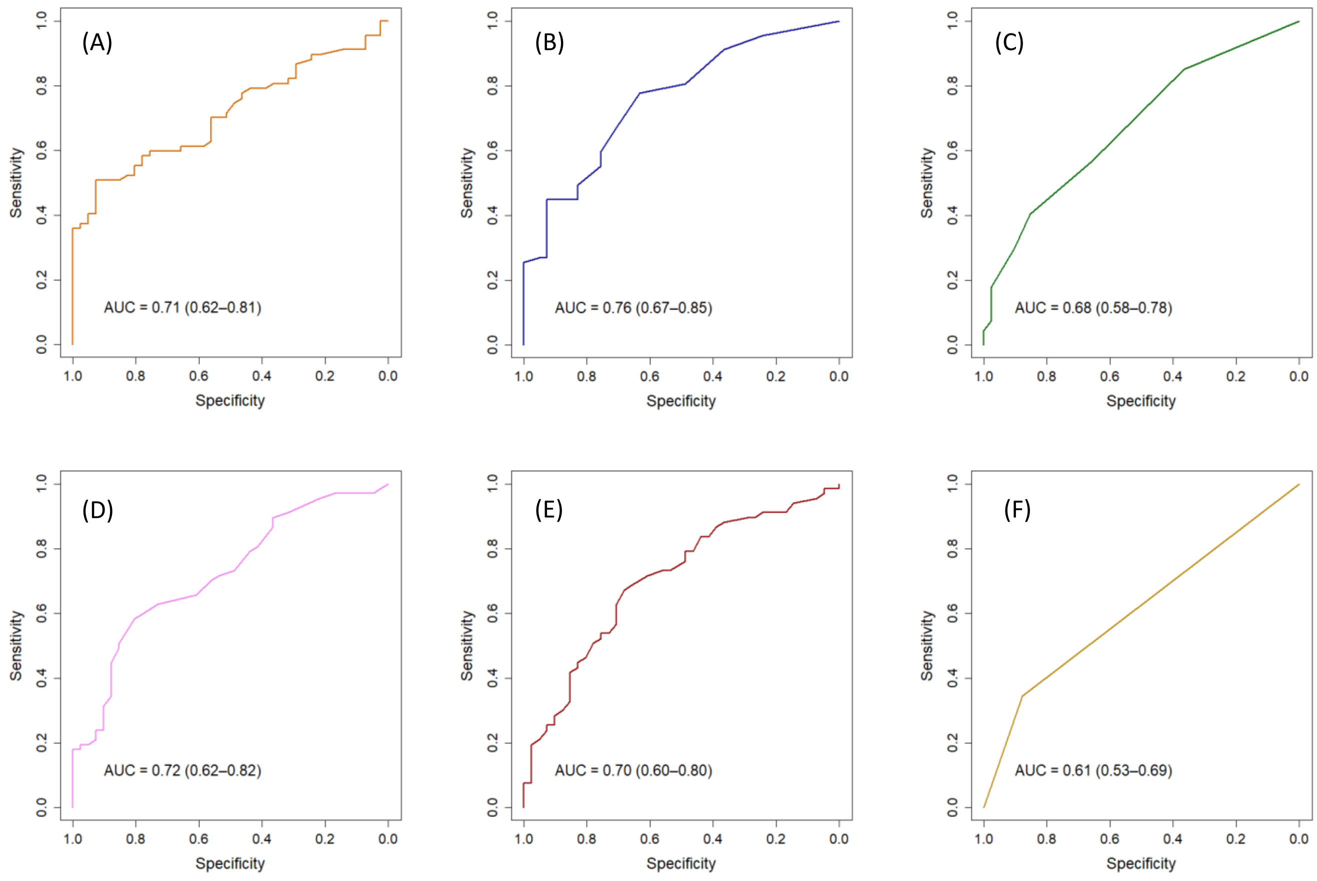

Supplementary Fig. S2 ROC curves for all subtests.
ROC curves depict TAP-Alertness (A), TAP-Sustained Attention (B), TAP-Divided Attention (C), TMT-A (D), TMT-B (E), and MoCA (F). MoCA = Montreal Cognitive Assessment; TAP = Test for Attentional Performance; TMT = Trail-Making-Test
